# Physiological Adaptation and Plant Distribution along a Steep Hydrological Gradient

**DOI:** 10.3390/plants11131683

**Published:** 2022-06-24

**Authors:** Kaj Sand-Jensen, Jens Borum, Claus Lindskov Møller, Lars Baastrup-Spohr

**Affiliations:** 1 Freshwater Biological Laboratory, Department of Biology, University of Copenhagen, Universitetsparken 4, 3rd Floor, 2100 København Ø, Denmark; ksandjensen@bio.ku.dk (K.S.-J.); jborum@bio.ku.dk (J.B.); 2 The Danish Environmental Protection Agency, Fejøgade 1, 4800 Nykøbing Falster, Denmark; cllin@mst.dk

**Keywords:** photosynthesis, functional traits, competition, hydrological gradient, biodiversity

## Abstract

Plant species often separate strongly along steep environmental gradients. Our objective was to study how coupling between plant physiology and environmental conditions shapes vegetation characteristics along a distinct hydrological gradient. We therefore investigated species photosynthesis in air and under water within a limited area from dry-as-dust to complete submergence in a nutrient-poor limestone habitat on Öland’s Alvar, Sweden. We found structural and physiological adaptations of species to endure water limitation at the dry end (e.g., moss cushions and CAM-metabolism) and diffusive carbon limitation (e.g., bicarbonate use) at the submerged end of the gradient. As anticipated, mean photosynthesis in air increased 18-fold from the species-poor assembly of cushion-mosses and *Sedum* CAM-species on mm-thin limestone pavements to the species-rich assembly of C-3 terrestrial plants in deeper and wetter soils. A GLM-model indicated that 90% of the variation in species richness could be explained by a positive effect of soil depth, a negative effect of the duration of water cover and their interaction. In water, mean photosynthesis was highest among aquatic species, low among *Sedum* species and cushion mosses, and negligible among C-3 terrestrial plants. While aquatic species dried out in air, drought-resistant small species were probably competitively excluded from the more suitable terrestrial habitats on deeper soils with moderate flooding by taller species of high photosynthetic capability. In conclusion, the clear distribution of species along the steep hydrological gradient reflects distinct structural and physiological adaptations, environmental filtering and interspecific competition.

## 1. Introduction

An important goal in plant ecology is to predict the occurrence and abundance of species inhabiting natural communities and examine the mechanisms behind their distribution [1]. Gradients of vital growth resources such as light, water and nutrients generate distinct distribution patterns of terrestrial species [2,3]. Topographically related soil wetness is the most important determinant of terrestrial habitat types on large scales such as across the entire 43,000 km^2^ in Denmark [4]. On local scales, hydrological gradients generate strong environmental filtering, distinct plant species distribution and marked trait selection as adaptations to either drought (e.g., low evapotranspiration and wilting point) or flooding (e.g., high root porosity) [5,6]. Niche segregation of species along hydrological gradients is common in dry as well as wet habitats [7,8,9,10]. Here, we examine plant species distribution in relation to physiological and structural adaptations along a steep hydrological gradient from dry-as-dust to permanent inundation within a small limestone area.

Aquatic plants usually have a thin leaf cuticle and rapidly dry out and die in air. Under water, light and concentration of dissolved inorganic carbon (DIC) are important for distribution and growth of aquatic plants [11,12]. Due to the 10,000 times slower diffusion of CO_2_ in water compared to air, the concentration of CO_2_ needed to saturate photosynthesis is often 7–30-times air-equilibrium concentrations [13]. Rapid photosynthesis can further deplete CO_2_ substantially below air-equilibrium concentrations. To alleviate carbon limitation, many submerged species have evolved biochemical mechanisms to use the dominant bicarbonate pool in fresh water (>95% of DIC at pH ~6.3–10.2), which allow them to extract 20–90% of the DIC pool instead of just a few percent if restricted to CO_2_ use alone [13,14,15]. Therefore, freshwater plants capable of using the large pools of bicarbonate have a strong competitive advantage compared to obligate CO_2_-users in waters close to air equilibrium of CO_2_ [16]. Concentrations of bicarbonate and CO_2_ vary extensively among individual water bodies and are followed by marked species distribution patterns [12,17,18].

When submerged, terrestrial C-3 angiosperms have difficulty maintaining photosynthesis because the stomata stop functioning for entry of CO_2_ in gas phase, the leaf cuticle restrains the inward CO_2_ flux through the main leaf surface and species lack mechanisms to use bicarbonate [19]. Terrestrial CAM-angiosperms may represent a special situation when submerged because they photosynthesize with closed stomata during daytime using CO_2_ from decarboxylation of malate produced by nocturnal CO_2_ uptake [20]. Mosses have leaves of one cell layer thickness and lack stomata as well as a cuticle to prevent dehydration. Mosses may photosynthesize quite well under water due to thin leaves of high gas permeability, despite their inability to use bicarbonate [14]. On land, photosynthesis of mosses is constrained by the loss of water in dry air. However, they tolerate water loss and rapidly become hydrated and resume photosynthesis when moisture or water becomes available [21]. Thus, we foresee that terrestrial plants and mosses photosynthesize under water at a rate depending on the species type and the CO_2_ concentration. Mosses may photosynthesize quite well under water at high CO_2_ concentrations, CAM-angiosperms likewise provided their malate pools have been built up before inundation, while C-3 angiosperms are expected to perform poorly.

In air, C-3 angiosperms photosynthesize at variable rates, with species growing on deeper soils with high resource supply of water and nutrients expected to have higher photosynthesis than species located on thinner soils with low resource supply. CAM-angiosperms and mosses are restricted to the lowest resource supply on very thin soils and are expected to have the lowest photosynthesis in air. Photosynthesis of terrestrial plants in air and aquatic plants under water has never been compared before across a hydrological gradient at the same site. We predict that terrestrial C-3 angiosperms, growing under high resource supply on thick soils, obtain the highest photosynthetic rates exposed to atmospheric CO_2_ because they are not constrained by the low diffusion rates of CO_2_ and bicarbonate meeting aquatic plants in water.

Both the photosynthetic performance in air and under water may influence the distribution of terrestrial plants across the hydrological gradient. One critical factor for survival of flooded land plants is oxygen supply to respiration which may be hampered by the low diffusion rates in water, the malfunction of stomata and the high cuticle resistance for oxygen uptake across the main leaf surface [22,23]. Another critical factor may be the required downward oxygen flux to root tips in anoxic sediments, which can be constrained by low oxygen production by photosynthesis and low root porosity (i.e., few air spaces) eventually leading to plant death [24]. Consequently, the transition between land and water forms a strong barrier for metabolism and survival of terrestrial as well as aquatic plants.

Our objective was to examine the coupling between plant physiology and distribution studying environmental conditions, vegetation characteristics and species photosynthesis in air and under water along the steep hydrological gradient from dry-as-dust to complete submergence in a nutrient-poor limestone habitat. Structural-functional species traits in combination with mutual ecological interactions may account for species distribution, which is accentuated due to the steep hydrological gradient. The specific hypotheses were, firstly, that photosynthesis is low among the small drought-tolerant species (e.g., succulent *Sedum* spp. and cushion-forming moss species) at the driest end of the hydrological gradient, but also relatively low among submerged species because of diffusive limitation of carbon supply at the opposite end of the gradient. While aquatic plants rapidly die in air, we hypothesize that terrestrial angiosperms have negligible photosynthesis under water because of an unsuitable leaf anatomy and physiology for the use of dissolved inorganic carbon. Secondly, we hypothesize that maximum photosynthesis and species richness may be found among medium-sized or tall land plants with leaves in air and roots in deeper soils or wet sediments providing a high combined air supply of CO_2_ and soil supply of water and nutrients.

## 2. Material and Methods

### 2.1. Site

The investigation was conducted in a limestone quarry on Räpplinge Alvar in Öland, SE Sweden (Baastrup-Spohr et al., 2015). The substratum consists of exposed solid limestone pavements, which are extremely dry and almost devoid of vegetation over large areas. Denser vegetation is found in crevices on the limestone plates. More permanent and richer vegetation grows in slightly deeper soils and sediments on the banks and within small ponds. The quarry is surrounded by natural alvar vegetation. The study section was abandoned 35 years ago and has been managed by horse grazing (2 horses on 6 ha), which has prevented encroachment by shrubs and trees. The site was selected because of the steep hydrological gradient with profound changes of species composition within a small area.

The climate is dry (mean annual precipitation 510 mm; 1960–1990), with moderately cold winters (January mean –1.2 °C) and mild summers (July mean 16.2 °C; [25]). On the exposed limestone pavements temperature exceeded 40 °C on 37 days (21%) between April and August [26]. Thus, the area has extreme gradients in water availability between water-filled ponds and exposed limestone pavements with mm-thin layers of dust [5]. The shallow ponds (0.1–0.8 m deep) fluctuate widely in depth, size and water chemistry among years and seasons.

Small shallow ponds are scattered throughout the area [27]. The alkalinity (=acid neutralizing capacity, ANC) in pond waters usually ranges from 0.8 to 2.0 meq L^−1^. Lower values occur after periods of heavy rainfall and higher values during late-summer because desiccation concentrates solutes [27,28]. Most ponds have a dense cover of characean macroalgae (charophytes) in active growth from April to June. The soils have low phosphorus concentrations (0.1–0.14 µg TP g DW^−1^) and the pond water has extremely low concentrations of both soluble inorganic nitrogen and phosphorus [28].

Along the gradients of water availability and soil depth, representative species are *Sedum album* and cushion-forming mosses at the driest places on thin soils or bare, solid limestone surfaces [27]. Some species (e.g., *Bromus hordaceus*, *Artemissia campestris* and *Festuca ovina*) grow in slightly deeper and wetter soils in crevices on the limestone plates. Many more species grow in deeper and wetter soils close to the ponds including *Alopecurus geniculatus* and species of *Carex* and *Juncus*. Large emergent plants such as *Alisma lanceolata*, *Alisma plantago-aquatica* and *Schoenoplectus taberneamontani* are found in shallow water at the fringe of the ponds, while the aquatic species *Myriophyllum spicatum*, *Zannichellia palustris* and *Chara* spp. grow totally submerged in the ponds.

### 2.2. Vegetation Sampling and Water Regime

A previous study examined community assembly and trait selection across the hydrological gradient in this location [5]. The study concluded that few plant traits are exposed to environmental filtering across the entire hydrological gradient, while most traits are strongly filtered only in parts of the gradient (e.g., root porosity in wet soils and water loss on drying on thin, dry soils). In order to determine the distribution of all species and those used for photosynthetic experiments as well as the environmental conditions they are exposed to, we used supplementary data on soil depth, number of days of flooding and characteristics of vascular vegetation from this previous study of twenty-one quadrates (0.5 × 0.5 m) placed by random stratified sampling. Plant species abundance was scored as presence or absence in each of 25 subplots (0.1 × 0.1 m) within each quadrat and species richness calculated from these data. Soil depth was measured in each subplot and number of days of flooding between mid-April and early June was calculated from measurement of elevation of the quadrats relative to continuous measurements of water level in four ponds in the study area (details in Baastrup-Spohr, Sand-Jensen [5].

In the new study, chemistry in eight ponds within the area was sampled at noon on a sunny day in late May and characterized by alkalinity, pH and dissolved CO_2_ using the same methods as when quantifying plant photosynthesis in water (see below).

### 2.3. Plant Photosynthesis and Other Key Traits

Photosynthesis was measured in air and in water on twenty-three species distributed along the hydrological gradient. In order to characterize the preferred habitat of species in terms of soil water supply, we applied individual Ellenberg F-values (Table S1). Ellenberg F-values is a classification of species on a 12-step scale from highly arid to fully submerged conditions according to extensive studies of their distribution in Central Europe [29].

For measurements of photosynthesis, healthy plant species were collected at the field site in whole turfs for terrestrial rooted species and entire cushions for mosses and transferred to plastic bags with a small water volume to keep the air humid and prevent desiccation. Submerged species were collected as individual shoots in water filled buckets. Photosynthesis was measured on terrestrial and submerged species within few hours after collection in the laboratory located 20 min drive from the sampling site. Moss cushions were placed outdoor for 24 h and kept wet to become activated before photosynthesis measurements. All measurements in air and in water were on three different specimens for every species. Photosynthetic rates were calculated per unit dry weight (DW, after drying in an oven at 105 °C). Mean values of the triplicates were used for subsequent calculations, data evaluation and illustrations.

Photosynthesis in air was measured on about the same combined weight of individual small leaves, parts of large leaves and small apical green shoots of mosses in a circulating air flow in a small transparent Perspex-chamber submerged in a cooling bath at 15 °C and illuminated by small halogen lamps at about 400 µmol photons m^−2^ s^−1^ (400–700 nm). The airtight tubes connecting the photosynthesis chamber and the infrared gas analyzer (LiCor, LI-840 CO_2_/H_2_O analyzer) were also submerged in the cooling bath to minimize temperature rise during measurements. Air temperature in the photosynthesis chamber was recorded continuously by a small Hobo-logger and was close to 20 °C (±1 °C). Measurements in light were preceded by dark measurements to attain equilibrium between leaf exchange and recordings of CO_2_ before light was switched on. Measurements were made close to air concentrations of CO_2_ (400 µatm). A wetted filter was placed in the chamber and measurements were kept short to avoid leaf desiccation and stomata closure. *Sedum acre* showed a slightly negative photosynthesis in air presumably because induced CAM-metabolism resulted in closed stomata and no net uptake of CO_2_ from air and was thus removed from analyses of aerial photosynthesis. Photosynthesis was calculated as the rate of decline of CO_2_ with time in the incubation chamber relative to dry weight of incubated plant material. Photosynthesis measured in air and under water in the light represents net photosynthesis, i.e., the net balance between gross production and respiration occurring in the light.

Photosynthesis in water was measured on the same type of leaf material in closed glass flasks (25 or 50 mL) mounted on a rotating wheel (12 rpm) in a temperature constant (20 °C) water bath and illuminated by fluorescent light tubes at about 400 µmol photons m^−2^ s^−1^ (experimental setup in Sand-Jensen et al. 1992) Two small glass beads were added to each flask to aid water mixing and prevent that build-up of stagnant water limits photosynthesis. Incubation water was collected from a large pond at the site, filtered and air-bubbled to attain air equilibrium of oxygen and CO_2_. Air bubbling was done indoor at about two-fold elevated air concentrations of CO_2_ resulting in the mean composition of the incubation water as: alkalinity 1.15 meq L^−1^, pH 8.0 and 33 µmol CO_2_ L^−1^. Photosynthesis was measured in three individual samples as oxygen evolution in flasks with plants relative to blanks in 2–3 h long incubation. At termination of the experiments, oxygen concentrations were measured by an oxygen microelectrode (OX-500, Unisense A/S, Aarhus, Denmark). Photosynthesis is the production rate of oxygen in the flasks relative to plant dry weight. In order to compare photosynthetic rates in air measured as CO_2_ uptake and under water measured as oxygen evolution, all rates were expressed as moles of carbon, using a molar photosynthetic quotient of 1.0. For the typically submerged species, *Ranunculus aquatilis*, both aquatic and aerial leaf types were present on the individuals at the sampling location and consequently aquatic leaves were used for photosynthesis in water and aerial leaves for photosynthesis in air.

In order to determine the ability of species to use dissolved inorganic carbon (DIC) in photosynthesis, we performed pH-drift experiments in illuminated, water-filled bottles with plants over 14 h driving pH to maximum levels and depleting DIC and CO_2_ to minimum concentrations where photosynthesis stops (Sand-Jensen et al., 2022) The experiments were performed using the same experimental setup as described above. Oxygen concentrations were reduced to 50% air saturation in the water before incubation to reduce subsequent oxygen accumulation and photorespiration. After incubation, 10.0 mL of water was withdrawn for acidimetric Gran titration [30] using 0.1 N HCl and continuous pH registration (Radiometer equipment, Copenhagen). Alkalinity and DIC were calculated from the titration and the CO_2_ concentration was calculated from end-pH, DIC and temperature according to Mackereth, Heron [31]. These experiments permitted calculation for each species of maximum attainable pH, minimum CO_2_ and extraction capacity of DIC (reduction of DIC relative to initial DIC, %) during extended photosynthesis. Species capable of using bicarbonate in photosynthesis can markedly deplete DIC and CO_2_ and attain high pH in contrast to sole CO_2_ users that can only deplete DIC marginally and yield pH and CO_2_ concentrations close to those in air saturated water (Sand-Jensen et al., 2022).

In order to describe the relationship between photosynthetic rates of species and their prevalence to water supply along the hydrological gradient, we related their photosynthesis to Ellenberg F-values [29]. The four cushion-forming moss species that grow together with *Sedum album* and *S. acre* under very arid conditions were assigned to the same individual Ellenberg F-value of 2. The mosses are, at least as drought resistant as the *Sedum* species. In contrast, the fully submerged charophyte, *Chara aspera* was assigned an Ellenberg F-value value of 12 used for all submerged species. Other species traits as adaptations to aridity and submergence included specific leaf area (SLA, cm^2^ g^–1^ DW) and root porosity (RP, air space in % volume). Thus, we related photosynthetic rates of species to those traits measured by Baastrup-Spohr, Sand-Jensen [5; their appendix 3].

### 2.4. Data Analyses

To evaluate how physiological and morphological traits change along the relative finely graded division of plant species into moisture preference groups (Ellenberg F-values) we used Spearman rank correlation due to the inherent character of the Ellenberg values (ordinal scale). Correlations between continuous traits were correlated using Pearson product moment correlation after evaluation of the assumption of normality.

Differences between plant groups with different overall photosynthetic and drought tolerance strategy (i.e., terrestrial C-3, aquatic C-3 and terrestrial CAM + mosses) in photosynthetic rates were tested by means of an ANOVA test followed by Tukey’s post hoc test. Deviance from homogeneity of variance was assessed by Brown-Forsythe’s test. We did not test the morphological trait differences between these groups, as many representatives of the aquatic C3 and CAM + mosses cannot be meaningfully evaluated for root porosity and SLA.

To evaluate how species richness was regulated along the investigated hydrological gradient we applied Poisson generalized linear models (GLM) with a log link function. The log link function ensures positive fitted values, and the Poisson distribution is commonly used for counts (such as species richness). The explanatory variables used were number of days with water cover and average sediment depth. Because the effect of sediment depth potentially depended on the water cover, we included an interaction term between the two variables in the model. The significance of the interaction term was evaluated by comparing nested models using analysis of deviance [32]. Check for overdispersion and model validation followed the procedure of Zuur, Ieno [32] and showed no indications of overdispersion. No trends in the residuals indicated violation of model assumptions.

Throughout, variables not normally distributed, according to the Shapiro–Wilk test, were log-transformed to ensure normality. Alpha levels below 0.05 were considered significant. Correlations, ANOVA tests and all graphs were performed in GraphPad Prism version 8.4.3 (GraphPad Software, San Diego, CA, USA) while the Poisson GLM were conducted using R version 4.0.2 [33] using the base package.

## 3. Results

### 3.1. Photosynthesis and Other Key Traits along the Hydrological Gradient

**Figure 1:**
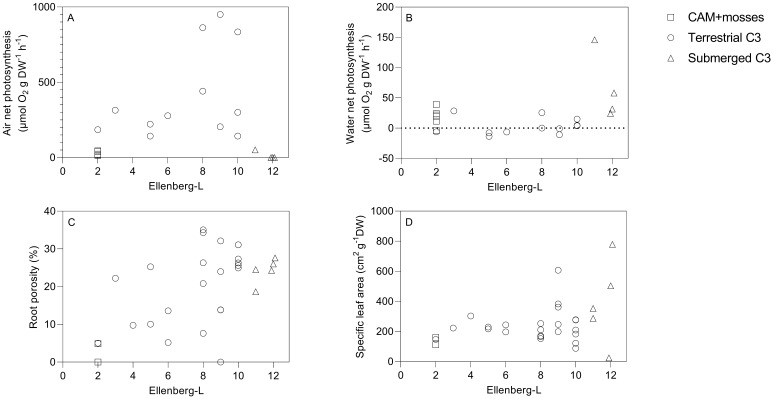
Species photosynthesis in air (**A**), photosynthesis in water (**B**) and functional traits (**C**): root porosity and (**D**): specific leaf area in relation to Ellenberg F-values along the hydrological gradient. Point shape indicate species group. Air photosynthesis of terrestrial species correlated highly significantly with Ellenberg F-values (*p* < 0.001). For all species there was a very significant positive relationship between Ellenberg F-values and root porosity (*p* < 0.01) and specific leaf area (*p* < 0.01).

As hypothesized, photosynthesis in air showed a marked peak for terrestrial species of intermediate Ellenberg F-values located in the middle of the hydrological gradient, while values were very low for species at the dry end and zero because of leaf desiccation of aquatic species at the submerged end of the gradient (Figure 1A). Mean photosynthesis in air was only 21 µmol C g^−1^ DW^−1^ h^−1^ and rates were systematically low among the four drought-resistant cushion mosses (range: 15–45 µmol C g^−1^ DW^−^ h^−1^) and the two succulent, constitutive CAM-species, *Sedum album* and *S. acre* (−4–39 µmol C g^−1^ DW^−^ h^−1^) of low Ellenberg-F indices (2) on thin soils as adaptations to arid conditions (Table 1). Mean photosynthesis was eighteen-fold higher (380 µmol C g^−1^ DW^−^ h^−1^) among thirteen terrestrial angiosperm species, including emergent plants in the shallow ponds, growing on thicker soils with better water supply (Ellenberg F indices: 3–10). Photosynthesis of terrestrial species in air increased highly significantly with their Ellenberg F index from 2 to 10 (Spearman Rank, r_s_ = 0.71, *p* < 0.001, *n* = 19) and higher specific leaf area (Pearson Product Correlation, r = 0.71, *p* < 0.02, *n* = 11). Submerged species (Ellenberg F of 11–12) rapidly desiccated when exposed to air [5] and, thus, did not photosynthesize.

In contrast, photosynthesis in water was significantly higher among the four submerged species compared with the negligible values among the thirteen terrestrial angiosperm C-3 species and the low values in the group of mosses and *Sedum* species (one-way ANOVA and Tukey’s post hoc test, *p* < 0.05, Figure 1B, Table 1).

Photosynthesis in water was low but positive in five of six cases among the mosses and *Sedum* species (Ellenberg-F = 2, Figure 1B), though not significantly different compared to photosynthesis of the thirteen terrestrial C-3 species (Table 1). In their natural freshwater environment, mean photosynthesis of submerged aquatic species was six-fold lower than terrestrial vascular C-3 species in air, but three-fold higher than mosses and *Sedum*-species in air (Table 1). Among the moss and *Sedum* species, mean photosynthesis was at the same low level in air and under water (Table 1).

The other key traits recorded at the species level also showed correlation with the Ellenberg F-values. Root porosity and Ellenberg F-values increased in concert and formed a highly significant positive relationship (Figure 1C, Spearman Rank, r_s_ = 0.54, *p* < 0.01, *n* = 29). Additionally, higher Ellenberg F-values were significantly correlated to increasing specific leaf areas (Figure 1D, Spearman Rank, r_s_ = 0.45, *p* = 0.01, *n* = 30).

### 3.2. Extraction of Inorganic Carbon in Water

The ability to raise pH, extract DIC and deplete CO_2_ below air saturation in water was very substantial for the four tested aquatic species, but minimal for the five terrestrial species (Table 2). The four aquatic species used bicarbonate and raised pH to 9.45–9.92, extracted 28–54% of the initial DIC pool and reduced CO_2_ between 25 and 120-fold below air equilibrium (i.e., 0.15–0.72 µmol L^−1^). The five terrestrial species did not raise pH and reduce CO_2_ much relative to air equilibrium and only extracted a minimum amount of the initial DIC pool (0.6–1.3%; Table 2). The difference between the two plant groups is so large and non-overlapping that statistical tests are not needed to infer the certainty of the observed differences.

In eight ponds in the area dominated by *Chara* spp., pH had reached 8.9–10.2 at noon and CO_2_ was markedly below air equilibrium (i.e., 0.06–2.7 µmol L^−1^; Table 3) precluding photosynthesis of terrestrial species.

### 3.3. Community Relationships

The large group of terrestrial C-3 species was widely distributed across the entire hydrological gradient, but most species was found at higher soil depth and low to intermediate water cover. Statistical analysis of species richness along the investigated hydrological gradient by means of a Poisson GLM regression showed a significant interaction between duration of water cover and sediment depth (*p* < 0.01, Table 4), while sediment depth alone had a significant positive effect on species richness (*p* < 0.01). This result indicates that while species richness increased with sediment depth this effect was dependent on the duration of flooding at the given site. This interaction led to the highest species richness being found on pond banks with deep soil and limited flooding. The explained deviance of the model, or pseudo R^2^ [32] was 89.9%, indicating that this high percentage of the variation in species richness could be explained by sediment depth, duration of water cover and their interaction.

The small CAM-species *Sedum album* did not at all occur in permanently inundated plots and its relative abundance increased significantly with decreasing soil depth in the remaining plots (Pearson R = −0.58, *p* = 0.046, *n* = 12). At the wet end of the hydrological gradient, the group of aquatic plants (Ellenberg F, 11–12) only occurred under permanently inundated conditions.

## 4. Discussion

The environmental conditions at the study site ranged from extremely dry with plants growing in soils few mm deep above the solid limestone plates and experiencing summer temperatures above 40 °C, through more productive areas with deeper soil, higher availability of soil nutrients and water in limestone crevices or close to ponds, to permanent submergence within the ponds. Most findings concur with expectations, but we have expanded the analysis to a complete hydrological gradient and have evaluated the distribution of species in relation to their photosynthetic capability both in air and under water.

Measured photosynthesis in air peaked at the mid to wet end of the hydrological gradient (Ellenberg-F: 8–10) among thirteen terrestrial C-3 species and, on average, dropped eighteen-fold to the four small cushion mosses and two *Sedum* species located at the arid end (Ellenberg-F: 2). We did not measure the growth rate of the twenty-three species, but may use photosynthetic rates as a proxy for this property [34]. It is likely that the growth rate of species follows the same distribution as photosynthesis being particularly low among species under the driest conditions and highest among the more numerous species in the mid to wet end of the gradient, where the vegetation is denser and taller [5]. The vegetation cover is very sparse on the open limestone plates where traits enabling survival during drought and restricted nutrient availability from thin soils are selected for whereas interspecific competition is minimal. The two small *Sedum* species have adapted to limited water availability by having small, densely packed succulent leaves and inducing CAM-metabolism under drought [20]. Their water loss on drying at 50 °C for 20 min was only 2–6% of the initial water content and, on average, ten-fold lower than the desiccation rates found among the terrestrial C-3 species located in the mid to wet end of the hydrological gradient [appendix 3 in 5].

The distribution pattern of *Sedum album* can be explained by a combination of high tolerance to limited water supply on thin soils and competitive exclusion by taller and faster growing species at higher nutrient and water supply in deeper soils. Field observations showed denser stands and greener shoots of *Sedum album* at sites with intermittent surface flow of water across the limestone surface and extra nutrients from horse droppings. These results support the notion that *Sedum album* and other drought-resistant small species are excluded from areas with deeper soils by competitive exclusion from taller species. The distribution pattern of *Sedum* species resembles that of other stress selected species of low competitive ability (*sensu* Grime [35]) such as *Salicornia europaea* confined to saline tidal flats [36] and small amphibious rosette species confined to wave-disturbed lake shores of low nutrient and organic content [37].

Mosses on the solid limestone surface form dense cushions reducing wind exposure and evaporation [21]. Moss cushions effectively suck up rainwater, retain it for several hours or days and tolerate subsequent desiccation. When rewetted, mosses rapidly restart photosynthesis and growth [38].

Under submerged conditions, species face two potential physiological problems. Both of them are caused by limited availability and diffusion rates of gases in water to support respiration and photosynthesis. Oxygen supply to respiration can be critical under water because oxygen concentrations are about 20-fold lower at air equilibrium and diffusion coefficients are 10,000-fold lower compared to air. Thin tissue, high surface permeability to gases, high tissue porosity and reduced respiration rates are the common anatomical and physiological adaptations to improve oxygen supply in response to submergence [22,23,24]. Among the tested species, we found that root porosity increased highly significantly under increasingly wet conditions according to Ellenberg F-values, thereby, supporting downward oxygen supply to roots buried in wet anoxic soils or sediments. The same increase of root porosity was found at the community level in quadrates of increasing wetness [5] pointing at root porosity as one of the most important selected traits in wet soils.

Supply of CO_2_ to underwater photosynthesis is similarly restricted by the lower diffusivity and, in addition, by CO_2_ concentrations reduced below air equilibrium, while concentrations required to saturate photosynthesis of submerged species based on CO_2_-use alone are some ten-fold above air equilibrium [13,16]. Even higher CO_2_ concentrations (i.e., 30 to 40-fold supersaturation) are required to saturate underwater photosynthesis of amphibious and terrestrial species [19]. Thus, in order to survive in the Alvar ponds with dense *Chara* vegetation, other submerged species need to use bicarbonate because photosynthesis in the examined ponds elevated pH and depleted CO_2_ between 7 and 360 times below air equilibrium at noon on summer days. These CO_2_ concentrations are close to or markedly below the CO_2_ compensation point of submerged species confined to CO_2_ use alone, limiting their photosynthesis and preventing growth [13,19]. The charophytes and angiosperms growing permanently submerged in the alkaline ponds assimilate CO_2_ by coupling bicarbonate use (HCO_3_^−^) to precipitating calcium carbonate (CaCO_3_) on their outer surfaces allowing continued photosynthesis without further rise of pH in the water (i.e., 2 HCO_3_^−^ + Ca^2+^ → CO_2_ (assimilated) + CaCO_3_ (precipitated); Sand-Jensen et al., 2021). Terrestrial species are excluded from the ponds, unless they can reach above the water surface, because they only extracted 0.6–1.3% of the DIC pool in the water and have CO_2_ compensation points above air equilibrium. The terrestrial species are unable to use bicarbonate and precipitate calcium carbonate (Sand-Jensen et al., 2022).

The ability of many submerged species to use bicarbonate and thrive under water comes with an investment cost to produce the necessary transport proteins and catalytic enzymes and running costs to operate the ion transport [39]. Likely because of this investment and the limiting inorganic carbon supply, we found that photosynthesis of submerged species, on average, was six-fold lower than terrestrial C-3 species in air. Other studies have confirmed that submerged species usually have much lower photosynthetic rates compared with terrestrial species under suitable resource supply because of lower availability of inorganic carbon and light under water [40]. A comprehensive study of eight *Chara*-species collected from alkaline ponds varying from oligotrophy to eutrophy reported mean photosynthetic rates (110 µmol O_2_ g^−1^ DW h^−1^) that were three-fold lower than mean rates of the terrestrial C-3 species from the oligotrophic limestone site [41].

In conclusion, we found highly distinct structural and physiological adaptations to endure water limitation at the dry end (e.g., CAM-metabolism of *Sedum* spp.) and diffusive carbon limitation at the submerged end (e.g., bicarbonate use) of the hydrological gradient in the limestone habitat. The species-rich assembly of terrestrial C-3 species was confined to better water and nutrient supply for roots in deeper and wetter soils as well as air contact for leaves in the mid to wet part of the hydrological gradient. These terrestrial C-3 species desiccated under drought and were unable to photosynthesize under water. Under optimal conditions, they benefitted from eighteen-fold higher mean photosynthesis compared to drought-resistant species and six-fold higher photosynthesis compared to submerged species. Drought-resistant species were likely competitively excluded from the more suitable habitats by taller and faster growing species. The distinct species distribution can be explained by the steep hydrological gradient, the specific structural-physiological adaptations combined with strong environmental filtering and interspecific competition. These relationships and interactions became highly distinct by examining species distribution and structural-physiological traits along a full hydrological gradient from dry-as-dust to full submergence.

## Figures and Tables

**Table 1:** Net photosynthesis (NP) in air and in water of four species of drought-resistant mosses and two constitutive CAM species of *Sedum* spp., thirteen C-3 terrestrial and emergent plants and four submerged species including the macroalga *Chara aspera*. Submerged species dry-out completely and die in air and have zero photosynthesis (0*). Mean values, SEM (standard error of the mean) and range are given. Different superscript letters depict significant differences between groups (one-way ANOVA, *p* < 0.05).

	NP in Air µmol C g^−1^ DW h^−1^			NP in Water µmol C g^−1^ DW h^−1^			
	Mean	SEM	Range	Mean	SEM	Range	*n*
Mosses and *Sedum* spp.	21.3 ^a^	7.9	−9.8–44.8	18.6 ^a^	5.8	−4.0–39.0	6
Terrestrial C-3 species	380 ^b^	84	63–950	1.2 ^a^	3.9	−16.6–28.5	13
Submerged species	0*	0*		64.9 ^b^	28.0	24–145	4

**Table 2:** Dissolved inorganic carbon (DIC) extraction capacity (%) and final CO_2_ of four submerged species and five terrestrial species in pH-drift experiments. Means, SEM (standard error of the mean) and range.

	Extraction Capacity of DIC (%)			Final CO_2_ (µmol L^−1^)			*n*
Leaf Type	Mean	SEM	Range	Mean	SEM	Range	
Submerged	46	6	28–54	0.34	0.01	0.15–0.72	4
Terrestrial	0.9	0.4	0.6–1.3	21.4	5.2	15.6–31.8	5

**Table 3:** Mean values and ranges of alkalinity, pH and CO_2_ concentration in eight ponds at the study site sampled at noon in late May when charophytes and submerged plants have high density.

	Mean	Range
Alkalinity (meq L^−1^)	0.97	0.78–1.21
CO_2_ concentration (µmol L^−1^)	0.82	0.06–2.7
pH	9.6	8.9–10.2

**Table 4:** Estimated regression parameters, standard error, z-values and *p*-value for the Poisson GLM model with species richness as the dependent variable and sediment depth, days with water cover and their interaction as explanatory variables.

	Estimate	SD	z-Value	*p*-Value
Intercept	1.96	0.31	6.43	<0.001
Sediment depth (cm)	0.22	0.067	3.23	0.0013
Water cover (days)	−0.0069	0.0079	−0.88	0.39
Sediment depth: Water cover	−0.0045	0.0014	−2.99	0.0027

## Data Availability

All data is supplied in the Supplementary materials.

## Supplementary Materials

The following are available online at https://www.mdpi.com/article/10.3390/plants11131683/s1, Figure S1: Species photosynthesis in air (A), photosynthesis in water (B), root porosity, specific leaf area and Ellenberg F-values along the hydrological gradient. Table S1: Net photosynthesis (NP) in air and in water of four species of drought resistant mosses and two constitutive CAM species, Table S2: DIC extraction capacity (%) and final CO_2_ of four submerged species and five terrestrial species in pH-drift experiments, Table S3: Alkalinity, pH and CO_2_ concentrations in eight ponds at the study site, Table S4: Poisson GLM model for species richness.
